# Purple Glove Syndrome: Recognizing a Rare Complication of Intravenous Phenytoin

**DOI:** 10.7759/cureus.23958

**Published:** 2022-04-08

**Authors:** Genesis Perez Del Nogal, Alyssa Rodaniche, Sailaja Devi Saragadam

**Affiliations:** 1 Internal Medicine, Texas Tech University Health Sciences Center, Odessa, USA; 2 Laboratory Science and Primary Care, Texas Tech University Health Sciences Center, Odessa, USA

**Keywords:** phenytoin, purple glove syndrome, neurology and systemic disease, adverse reactions, drug-related side effects and adverse reactions

## Abstract

An uncommon but serious adverse drug reaction after phenytoin administration is known as purple glove syndrome (PGS). Initial presentation is characterized by pain, skin discoloration, and edema, that can progress to necrosis. The pathophysiology remains uncertain; however, multiple mechanisms have been reported including extravasation. We describe a case of a 61-year-old patient who was brought to the hospital with altered mental status due to status epilepticus. The patient received multiple doses of lorazepam; eventually was started on levetiracetam and valproate, including loading doses. The seizures were poorly controlled despite treatment, and intravenous (IV) phenytoin was added. The next day, bluish discoloration and swelling to bilateral upper distal extremities were noted on physical examination. Consequently, IV phenytoin was discontinued immediately due to high suspicion of PGS. Skin discoloration and edema gradually improved after one week, confirming a case of mild PGS.

## Introduction

Purple glove syndrome (PGS) is a rare severe adverse drug reaction typically associated with intravenous (IV) phenytoin administration. It is characterized by purple-bluish discoloration around the IV site, peripheral edema, and pain. The etiology is unknown; nonetheless, several risk factors have been associated with PGS, such as elderly patients and patients receiving large, and multiple doses of phenytoin [1]. According to a prospective study on the incidence of PGS, the incidence was determined to be 1.7% compared to the published rate of 5.9% [2]. Here, we present a case of a woman with mild symptoms of purple glove syndrome following IV phenytoin administration and discuss the pathophysiology of this rare complication, management, and preventive measures.

## Case presentation

A 61-year-old female presented to the hospital with altered mental status due to status epilepticus. Her medical history was significant for hypertension and coronary artery disease. On evaluation, she was somnolent and confused, but able to follow simple commands without focal neurological deficits. The patient denied any history of seizures, recent trauma, loss of consciousness, headache, dizziness, nausea, vomiting, pain, shortness of breath, diarrhea, or dysuria. There were no significant findings on the physical examination except for the neurologic status as described above. At the emergency department, the patient received multiple doses of lorazepam and was started on levetiracetam 1.5 gram (g) twice daily.

**Figure 1:**
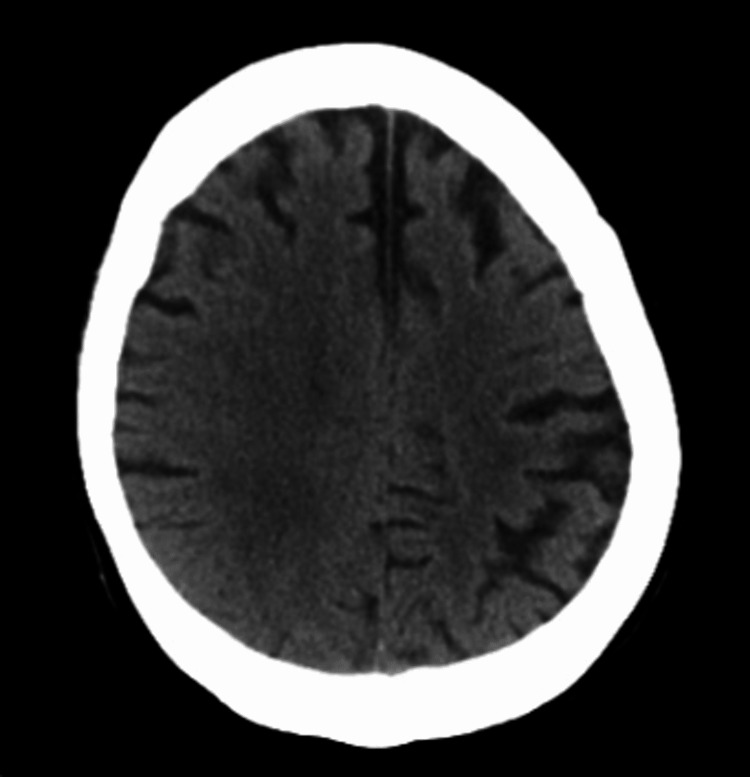
CT scan of the head revealed diffuse parenchymal volume loss. CT: computerized tomography

**Figure 2:**
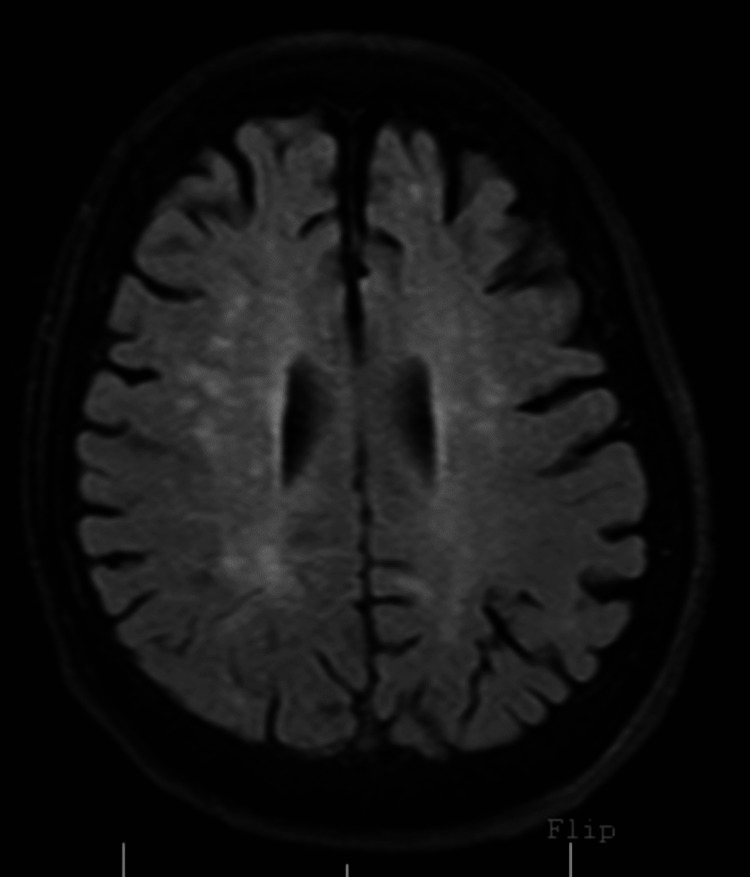
MRI brain showed moderate chronic microvascular ischemic changes. MRI: magnetic resonance imaging

Laboratory studies on admission were significant for leukocytosis (13x10^3^/μL, neutrophils %: 86.5%), and the complete metabolic panel showed borderline hypokalemia (3.4 mmol/L). Urinalysis and urine drug screen were negative. Computerized tomography (CT) scan of the head revealed diffuse parenchymal volume loss (Figure 1). Magnetic resonance imaging (MRI) of the brain did not show any acute infarct or hemorrhage, only moderate chronic microvascular ischemic changes appeared (Figure 2).

**Figure 3:**
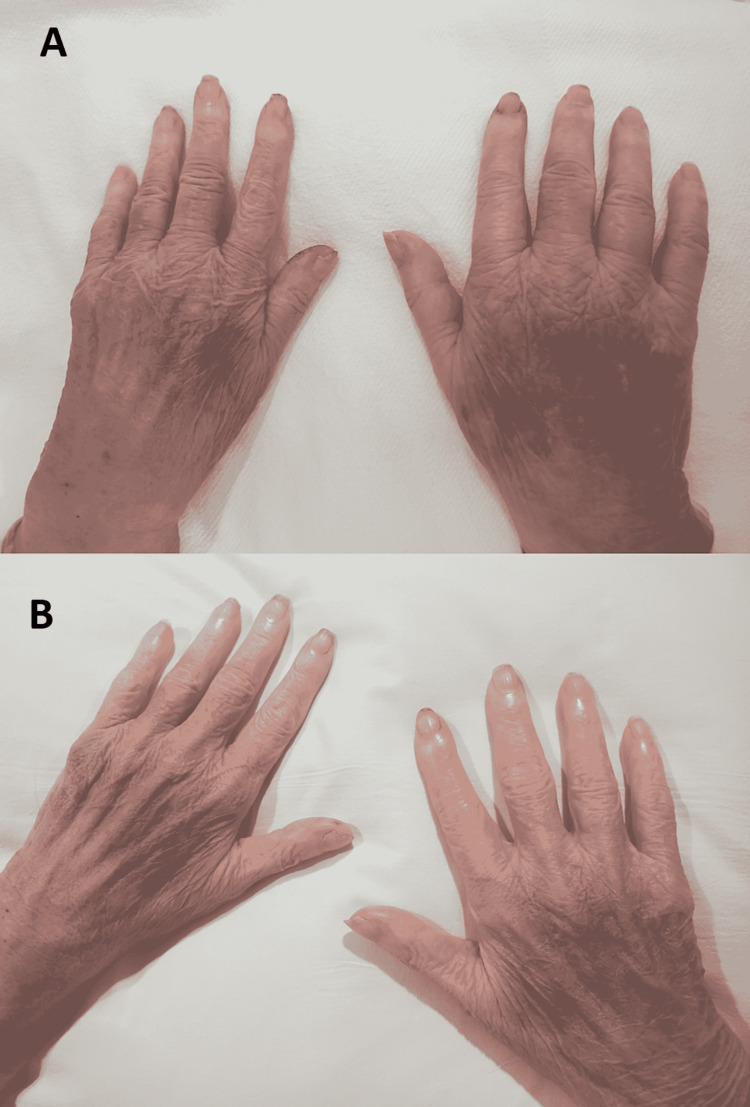
The images show (A) bluish discoloration and swelling of both hands (one day after IV phenytoin) and (B) partial resolution of swelling and skin discoloration (two days after phenytoin was discontinued). IV: intravenous

The seizures were poorly controlled despite treatment, valproate 500 milligrams (mg) every eight hours was started and neurology consultation was obtained. During her hospital course, electroencephalogram showed epileptiform discharges and lumbar puncture was negative for infection. The patient still developed generalized tonic-clonic seizures, the neurologist decided to increase the dose of levetiracetam to 2 g twice daily and added phenytoin 100 mg IV every eight hours. After two doses of phenytoin IV, bluish discoloration and swelling on bilateral upper distal extremities were noticed (Figure 3).

Due to high suspicion of PGS, IV phenytoin was discontinued immediately and switched to lacosamide. PGS was managed with upper limbs elevation, accompanied by heat and cold application. The patient’s skin discoloration and edema gradually improved after one week, confirming a case of mild PGS. Furthermore, seizures subsided, and we were able to titrate antiseizure medication without recurrence of seizures.

## Discussion

Purple glove syndrome (PGS) is a rare severe adverse drug reaction typically associated with intravenous (IV) phenytoin administration. PGS is poorly understood and only 82 cases were reported between 1984 and 2015 [3]. Symptoms of PGS include dark purple-bluish discoloration around the site of IV phenytoin infusion, peripheral edema, and pain. On rare occasions, PGS may progress to compartment syndrome, necrosis, ischemia, or vascular compression [4,5].

The true pathophysiology of purple glove syndrome is not fully understood; however, it is believed to be due to chemical irritation secondary to the high alkalinity of phenytoin and propylene glycol. The alkaline solution could stimulate vasoconstriction and thrombosis, producing extravasation into the surrounding interstitium [6,7].

According to data from two institutions, the incidence of PGS after parenteral phenytoin is 1.7-5.9% [2]. Those aged 60 years and older, female sex, sepsis, peripheral vascular disease, history of chronic debilitating disease, and those who receive larger and more frequent doses of IV phenytoin are at increased risk of PGS [1,4,8].

Here, we described a female patient with a past medical history of hypertension and coronary artery disease, who presented to the hospital with status epilepticus. The patient received multiple doses of lorazepam and levetiracetam. The seizures were not controlled despite treatment, and valproate was started. However, the patient continued to have generalized tonic-clonic seizures and phenytoin was started as per neurology recommendations. The day after, bluish discoloration and edema were evidenced on bilateral upper distal extremities. Phenytoin was discontinued immediately due to high suspicion of PGS, and the patient was switched to lacosamide.

Several published case reports noted improvement of mild cases of PGS with supportive measures, such as discontinuing IV phenytoin, limb elevation, and heat or cold application, as in our case. Resolution varied from days to weeks [1,3,9]. On the other hand, severe cases may require emergency surgical intervention, like fasciotomy, to relieve pressure and restore blood flow [4,5]. In conclusion, clinicians should be aware of this uncommon adverse reaction of IV phenytoin and should discontinue the drug promptly if the syndrome is suspected to avoid further complications.

## Conclusions

This is a classic case of purple glove syndrome and early recognition is essential to minimize the morbidity of this rare adverse drug reaction. We recommend using alternative anticonvulsant medications in patients with increased risk for PGS. Effective teamwork between multidisciplinary team members, including neurologists and hospitalists, is crucial to achieving prompt diagnosis and optimal recovery. A high level of suspicion must be taken to avoid this rare but preventable syndrome.
